# Public perceptions of eye symptoms and hospital services during the first UK lockdown of the COVID-19 pandemic: a web survey study

**DOI:** 10.1136/bmjophth-2021-000854

**Published:** 2021-10-13

**Authors:** Gibran F Butt, James Hodson, Graham R Wallace, Saaeha Rauz, Philip I Murray

**Affiliations:** 1 Academic Unit of Ophthalmology, Institute of Inflammation and Ageing, University of Birmingham, Birmingham, UK; 2 Medical Statistics, University Hospitals Birmingham NHS Foundation Trust, Birmingham, UK; 3 Ophthalmology, Sandwell and West Birmingham Hospitals NHS Trust, Birmingham, UK

**Keywords:** COVID-19, infection, public health

## Abstract

**Objective:**

This study aimed to explore the British public’s healthcare-seeking beliefs concerning eye symptoms, and assess how the first COVID-19 lockdown influenced these.

**Methods and analysis:**

An anonymous web-based survey was disseminated through mailing lists and social media between June and August 2020. The survey sought participants’ views on the severity and urgency of the need for medical review for four ophthalmic and two general medical scenarios on a five-point scale. Participants were asked to answer questions twice: once ignoring the COVID-19 pandemic, and once taking this into account, with additional questions asked to identify factors influencing the decision to seek medical attention and ward admission.

**Results:**

A total of 402 participants completed the survey (mean age 61.6 years, 63.1% female and 87.7% of white ethnicity). Scores for symptom severity and urgency of medical review increased significantly with the severity of the clinical scenario (both p<0.001). However, participants gave significantly lower scores for the urgency of medical attention when accounting for the COVID-19 pandemic (compared with no pandemic) for all scenarios (all p<0.001). Younger age, greater deprivation and non-white ethnicity were correlated with a lower perception of seriousness and urgency of medical attention.

**Conclusions:**

During the first UK lockdown of the COVID-19 pandemic, reduced urgency of medical review for ocular and systemic pathologies was reported in response to the pandemic, which represents a barrier to healthcare-seeking behaviour. This has the potential to critically delay medical review and timely management, negatively impacting patient outcomes.

**Key messages**

**What is already known about this subject?**

- The COVID-19 pandemic has adversely impacted patient attendance to ophthalmic services.
- Although eye health is of high importance to the public, knowledge of eye problems is comparatively low.

**What are the new findings?**

- Survey participants were able to appropriately differentiate clinical scenarios of differing severity and seriousness.
- Symptoms consistent with microbial keratitis were considered to be as serious and impactful as those of angina and bowel cancer.
- For a given set of symptoms, participants indicated a lower likelihood of seeking eye care during the COVID-19 pandemic, compared with normal circumstances.

**How might these results change the focus of research or clinical practice?**

- The change in public healthcare-seeking behaviour during the first national lockdown in the UK has identified the importance of public health literacy in their utilisation of eye care services, and the need to accurately assess the clinical impact of this behavioural adaptation.

## Introduction

During the early stages of the COVID-19 (SARS-CoV-2) pandemic in the UK, attendances to emergency departments (ED) and emergency admissions decreased from 1 969 691 in February 2020 to 916 581 in April 2020,^1^ despite the Academy of Medical Royal Colleges,^2^ and the UK government prompting people to continue seeking medical help where necessary.

A similar and equally alarming pattern has been described for the ophthalmic ED, where reductions in attendances have been reported.^3–11^

The ED at the Birmingham and Midland Eye Centre (BMEC) is a major ophthalmic ED for the West Midlands, which records approximately 120 patient attendances a day, comprising community, secondary care, as well as walk-in self-referrals. Emergency and urgent referrals constitute a critical part of the ophthalmic service^12^; however, due to the COVID-19 pandemic, there was a significant reduction in face-to-face appointments and an increase in telephone consultations at the BMEC ED, in efforts to safely minimise patient-patient and patient-staff exposure.^13^ Each year approximately 100 emergency admissions are made to the BMEC for microbial keratitis (MK),^14^ which constitutes the most common non-surgical emergency and indication for admission in eye care services.^15^ Prompt management of MK is essential as, if untreated, MK can rapidly lead to profound irreversible sight loss.^16^

Rosenstock’s health belief model describes how the act of seeking help is consequent to the interplay of core factors, including the individual’s perceptions of disease severity, susceptibility, benefits and barriers to action.^17^ While the driving factors (severity of disease and benefits of seeking help) must outweigh the barriers to action for healthcare seeking to occur, prompt symptom recognition underpins timely engagement with health services. While the general public consider eye health to be critical to overall health,^18^ knowledge about eye disease and eye care services is poor,^19–22^ which could contribute to a misunderstanding of risk. As such, the pandemic may be considered a barrier to seeking care in Rosenstock’s health belief model.

Amidst concerns about the change in public healthcare-seeking behaviours and service utilisation, this study aimed to explore the public’s perception of eye symptoms, self-management strategies, hospital services and clinical research, and to better understand how the pandemic has affected healthcare-seeking behaviour. The Care Quality Commission confirmed that research is a priority for improving patient care that is embedded in the well-led framework^23^; changes in patient attitudes regarding participation in research will disrupt recruitment and delivery of future clinical research. Such changes may result from the public’s concern about the greater risk of exposure at healthcare facilities.^24 25^ Moreover, news of poorer outcomes in the elderly and ethnic minority patients with COVID-19^26 27^ has heightened anxieties in these individuals.^28–30^ Thus, the pandemic’s influence on health and healthcare services is widespread and disproportionally impacts some members of society.

## Methods

This cross-sectional study was conducted between June and August 2020 using an anonymous online survey to collect opinions of the first national lockdown in the UK. The study adhered to the principles of the Declaration of Helsinki. A proportionate approach to consent was adopted, and participants taking time to complete the questionnaire were deemed to have provided implied consent.

The open survey, available in English, was administered through the web tool Research Electronic Data Capture V.9.6.3 (Vanderbilt University, Nashville, Tennessee, USA), and a convenience sample was obtained through dissemination via social media (Facebook, Twitter, Instagram and LinkedIn); University of Birmingham and local community group mailing lists; general practice patient participation groups from around England; and the 1000 Elders research participation group, University of Birmingham, UK. A short video, produced using Doodly (Bryxen, Ohio, USA), accompanied and explained the survey (online supplemental video S1, https://www.youtube.com/watch?v=a-42fFn5meQ&feature=youtube).

10.1136/bmjophth-2021-000854.supp1 Supplementary video

The survey was refined through multiple rounds of testing for face validity by clinical and lay volunteers, who provided feedback on language, content, style and length.^31^ The complete survey can be accessed in the supporting information (online supplemental survey S2), and is summarised below.

10.1136/bmjophth-2021-000854.supp2 Supplementary data

Demographic data were collected, namely: gender, age (as ranges, eg, 18–25 years), ethnicity, occupation and postcode. Individuals were categorised by their occupation into either the ‘working’ group (in full-time or part-time education or employment) or ‘not-working’ group (retirees, homemakers, unemployed and individuals not working due to health reasons). Postcodes were used to determine the Index of Multiple Deprivation (IMD) score using the 2019 English and Welsh government data.^32 33^ The IMD score is based on seven domains: income, employment, health deprivation and disability, crime, barriers to housing and services, and living environment deprivation. For partially completed postcodes (eg, SW1A), an averaged IMD was derived from all the corresponding postcodes. The national deciles of the resulting scores were used for further analyses, with decile 1 being the most and 10 the least deprived.

Section I asked four questions about six hypothetical clinical scenarios (table 1) that were answered on a 5-point Likert scale (Not at all, Not very, Somewhat, Moderately, Very; scoring 1–5 points, respectively). The first two questions asked about the seriousness of the scenario, and how it would impact daily life. The other two questions asked how quickly participants would seek medical attention for the scenario, both if the COVID-19 pandemic was not a factor, and after taking the COVID-19 pandemic into account.

**Table 1:** Summary of clinical scenarios

Potential diagnosis	Description of scenario given in survey
Scenario 1—eye mildly red and gritty	
Dry eye disease	Over the previous week you have noted that your right eye feels gritty as though you have sand in it. The eye looks minimally red, it is not sticky and your vision is unaffected. You have not experienced these symptoms before.
Scenario 2—eye red, sticky and blurred	
Conjunctivitis	Over the previous week your right eye is red and sticky. It is slightly uncomfortable and your vision is slightly blurred, but not all the time. You have not experienced these symptoms before.
Scenario 3—eye red, painful, photophobia, sticky, blurred, white spot	
Microbial keratitis	Over the previous 2 days your right eye is red, painful and sensitive to light. It is sticky and your vision is blurred. You also notice there is a white area on your eye. You have not experienced these symptoms before.
Scenario 4—painless loss of vision	
Retinal detachment/retinal vascular occlusion	Over the previous day the vision in your right eye becomes very blurred. The eye is NOT red, painful or sticky. You have not experienced these symptoms before.
Scenario 5—rectal bleeding	
Bowel cancer	Over the previous week you visit the bathroom and notice that there is blood in your stools. This has happened several times over the last couple of weeks. Recently, you’ve been going to the toilet more often and have had some diarrhoea. You have also noticed that you have been losing weight, which is unusual because your appetite has been normal, and you have not been exercising more than normal. You are also feeling run down and very tired.
Scenario 6—chest pain	
Angina	Over the previous week whenever you undertake physical activity you experience a pain across your chest. The pain feels like a heaviness and tightness in the chest area. You also experience light-headedness and a slight shortness of breath. The symptoms subside after a few minutes, but start again when you engage in strenuous activities or when you experience emotional upset and stress.

Scenarios 1–3 represented combinations of ocular surface disease symptoms of progressively increasing severity. Scenario 1 symptoms were typical of mild dry eye, scenario 2 was consistent with conjunctivitis and scenario 3 with MK. Scenario 4, painless loss of vision, was consistent with multiple differential diagnoses requiring urgent review (eg, retinal detachment or retinal vascular occlusion). Two additional overtly serious non-ophthalmic scenarios were included to benchmark the ophthalmic scenario responses against conditions that participants could identify as serious and requiring urgent medical attention.^34^ Accordingly, scenarios 5 and 6 described symptoms of rectal bleeding (consistent with bowel cancer) and chest pain (consistent with angina), respectively.

Section II explored scenario 3 (MK) further by evaluating the influence of six factors on healthcare-seeking ideation, and of five factors on the decision to agree to hospital admission. A 5-point Likert scale was used (Strongly agree, Agree, Somewhat agree, Disagree, Strongly disagree; scoring 1–5 points, respectively). An additional set of questions inquired about the likelihood of using seven different self-management strategies for scenario 3.

Section III asked participants if they had a contact (family/friend/work colleague/other, where ‘other’ may be in any capacity, eg, at work in a clinical setting) with the conditions discussed in the survey. Further questions asked about the sources of information that participants would access about the COVID-19 pandemic, and before seeking an ophthalmologist. In each case, participants ranked their top three sources from a list (see online supplemental survey S2).

Section IV asked whether participants would be willing to volunteer for an ophthalmic research study, both if the COVID-19 pandemic was not a factor, and after taking the COVID-19 pandemic into account.

Returned surveys where section I was incomplete were excluded from analysis. For the remaining sections, where questions were not completed, participants were excluded from analysis for that specific question.

### Statistical analysis

Due to the established differences of COVID-19 impact and outcomes in the ethnic minority community, comparisons were initially made between participants of white and non-white ethnicities. The aim of this comparison was to test for any differences in demographics between ethnicities, which may have acted as confounders in the analysis, as well as to test how perceptions varied with ethnicity. These comparisons were performed using Fisher’s exact tests for nominal variables and Mann-Whitney U tests for ordinal variables.

Responses to questions were then compared across the six scenarios using Freidman’s test, followed by post hoc pairwise comparisons, where applicable. For each of the scenarios, questions that were answered with COVID-19 both being and not being a factor were compared using Wilcoxon signed-rank tests. Spearman’s rank correlations were used to describe the relationship between the answers to section I and the demographic factors: age, ethnicity, IMD, occupation and gender. All analyses were performed using IBM SPSS V.22 (IBM), with p<0.05 deemed to be statistically significant.

### Patient and public involvement

Patient and lay volunteers were engaged in the development of the survey, as well as assisting in the dissemination of the survey by means of sharing the web link and video. Once the study is published, the results will be disseminated through the same channels as the original survey.

## Results

### Cohort characteristics

Over the 8 weeks the survey was accessible, 524 responses were generated, of which 402 completed section I and so were included in the analysis. Participants were predominantly female (253; 63.1%) and of white ethnicity (348; 87.7%), with a mean age of 61.6 years. Most participants were retired (60.4%), with 36.6% in either employment or education. The postcodes provided by participants were distributed around England, although with a larger number around the University of Birmingham, BMEC and the surrounding areas (online supplemental figure 1). Consequently, participants were from the full range of IMD deciles, with a preponderance around the midpoint (17.8% in decile 5). Over half of the cohort knew someone who had COVID-19 (57.7%), with the majority also knowing someone with the diseases described in the questionnaire (eye disease, bowel cancer, angina; table 2).

10.1136/bmjophth-2021-000854.supp3 Supplementary data

**Table 2:** Cohort characteristics

	Total n	n (%)
Gender (% female)	401	253 (63.1)
Age (years)	400	*Mean*: 61.6*
18–25		24 (6.0)
26–35		45 (11.3)
36–45		18 (4.5)
46–55		20 (5.0)
56–65		49 (12.3)
66–75		159 (39.8)
76+		85 (21.3)
Ethnicity	397	
White		348 (87.7)
Asian or Asian British		33 (8.3)
Black or Black British		7 (1.8)
Mixed		5 (1.3)
Other		4 (1.0)
Employment	402	
Full-time employment		89 (22.1)
Part-time employment		35 (8.7)
In education		23 (5.7)
Retired		243 (60.4)
Home maker		7 (1.7)
Not working due to illness/disability		3 (0.7)
Unemployed		2 (0.5)
IMD decile	371	
1 (Most deprived)		23 (6.2)
2		15 (4.0)
3		37 (10.0)
4		47 (12.7)
5		66 (17.8)
6		38 (10.2)
7		40 (10.8)
8		36 (9.7)
9		38 (10.2)
10 (Least deprived)		31 (8.4)
Do you know or have known someone with the following conditions?		
Eye disease	402	264 (65.7)
Bowel cancer	402	207 (51.5)
Angina	402	233 (58.0)
COVID-19	402	232 (57.7)

Total n represents the number of participants who answered the stated question.

*The mean age was estimated by assigning each participant to the midpoint of the age range that they had specified.

IMD, Index of Multiple Deprivation.

Due to the established differences of COVID-19 impact and outcomes in the ethnic minority community, comparisons of the white (87.7%; n=348) and non-white (12.3%; n=49) subgroups were performed (online supplemental table 1). This found white participants to be significantly older (mean 65.1 vs 36.5 years, p<0.001) and, hence, more likely to be retired (p<0.001). White participants were also significantly more likely to be female (65.2% vs 49.0%, p=0.039) and had lower levels of deprivation (16.1% vs 53.8% in IMD deciles 1–3, p<0.001). Both groups were similarly likely to know contacts with eye disease (p=0.264). White participants were significantly more likely to know contacts with a history of bowel cancer (55.7% vs 22.4%, p<0.001) and angina (60.9% vs 40.8%, p=0.009), while non-white participants were more likely to know contacts with a history of COVID-19 (77.6% vs 55.2%, p=0.003).

10.1136/bmjophth-2021-000854.supp4 Supplementary data

### Seriousness of symptoms

**Figure 1:**
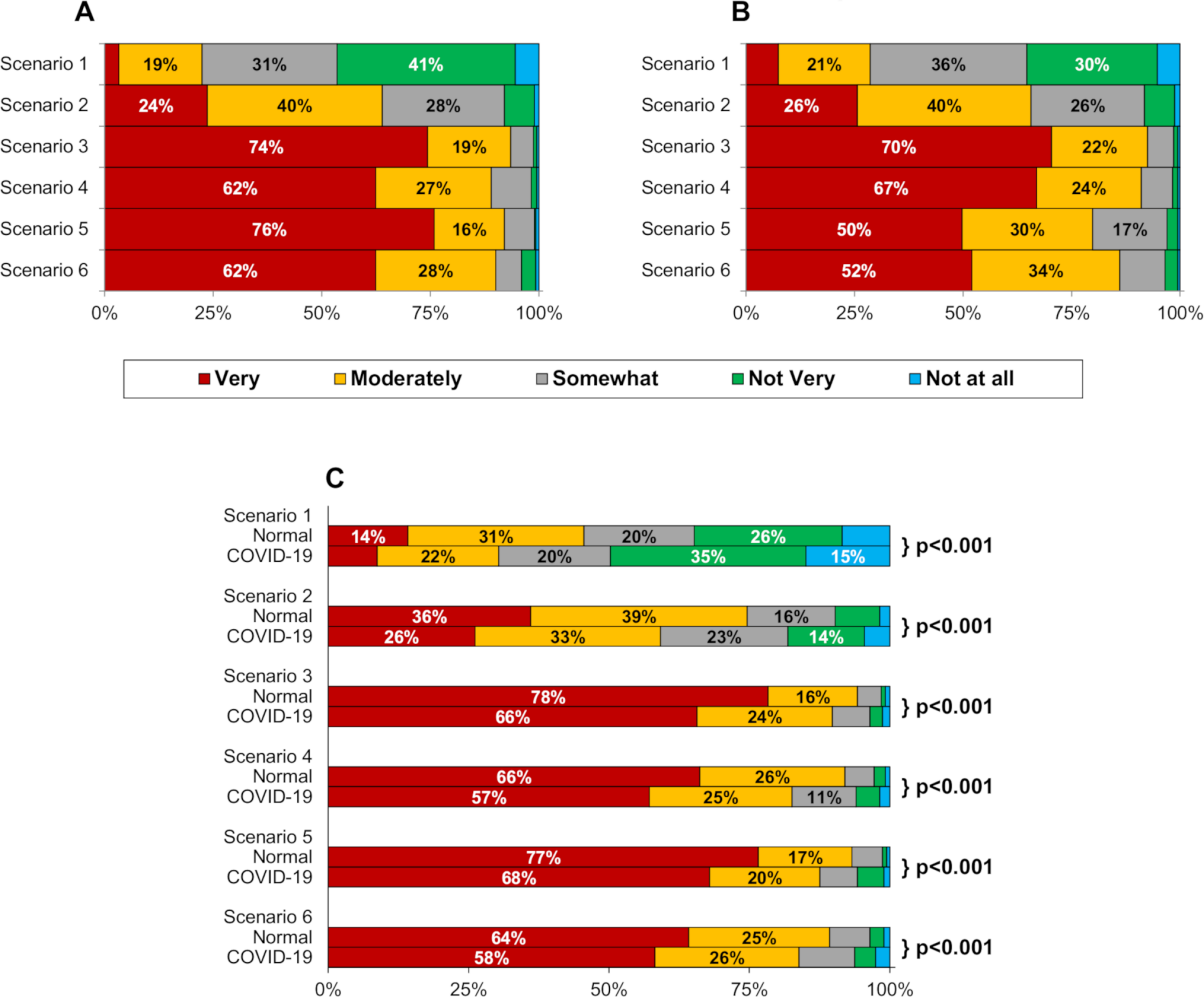
Clinical scenarios perceived seriousness, impact and urgency of medical attention. Scenarios are as described in table 1. For each scenario, participants were asked to indicate how serious (A) and impactful (B) the symptoms would be, and how quickly they would seek medical attention (C) if the COVID-19 pandemic was not currently a factor (‘Normal’), and after taking into consideration the COVID-19 pandemic (‘COVID-19’). For all three figures, responses were found to differ significantly across the six scenarios (Friedman’s test, p<0.001 in each case). In (C), p values are from Wilcoxon signed-rank tests, comparing Normal versus COVID-19 for each scenario. Unlabelled bars each consist of <10% of participants.

The reported seriousness differed significantly between the six scenarios (p<0.001, figure 1A). A progressive increase in the severity score was observed over the first two scenarios (1=dry eye disease, 2=conjunctivitis), with 90 (22.4%) and 257 (63.9%) of participants, respectively, reporting these as ‘moderately’ or ‘very’ serious. Scenario 3 (MK) was reported as moderately/very serious by 376 (93.5%), which was similar to the 358 (89.1%), 370 (92.0%) and 362 (90.0%) in the more overtly serious scenarios: 4=painless loss of vision, 5=bowel cancer and 6=angina, respectively.

### Impact of symptoms

Responses about the impact of the symptoms on daily life demonstrated similar trends to those described for seriousness (p<0.001, figure 1B), with 115 (28.6%), 264 (65.7%) and 372 (92.5%) participants rating the impactfulness as moderately/very for the first three scenarios, respectively. The response to scenario 4 was similar to that for scenario 3, with 366 (91.0%) stating this to be moderately/very impactful (p=1.000). However, symptoms of both scenarios 3 and 4 were reportedly more impactful than scenarios 5 and 6 (bowel cancer and angina), for which 321 (79.9%) and 346 (86.1%) responded moderately/very (all pairwise comparisons, p<0.001).

### Urgency of medical attention

Similar to the previous questions, a progressive increase in urgency across scenarios 1–3 (all pairwise comparisons, p<0.001), and comparable responses for scenarios 3–6 were observed (all pairwise comparisons, p>0.05). This was true for responses both during the pandemic and during ‘normal’ conditions (figure 1C). Within each scenario, participants rated urgency significantly lower when asked to take the COVID-19 pandemic into consideration (all p<0.001). For example, in scenario 3 (MK), the proportion of participants rating urgency as ‘very’ fell from 78.4% to 65.7% when taking the COVID-19 pandemic into consideration.

The full Likert score results for all scenarios are available in online supplemental table 2.

10.1136/bmjophth-2021-000854.supp5 Supplementary data

### Associations of age, gender, ethnicity and deprivation

Responses were compared across demographic factors (online supplemental table 3). The strongest associations were observed in scenarios 5 and 6 (bowel cancer and angina), where older participants tended to rate seriousness, impact and urgency more severely. Non-white participants and those from more deprived backgrounds tended to give lower scores for these outcomes. Subgroup analysis of the non-white participants found the relationship between deprivation and the reporting of lower seriousness, impact and urgency of symptoms to be stronger than for the cohort as a whole. No significant associations with age were observed in this subgroup (online supplemental table 4).

10.1136/bmjophth-2021-000854.supp6 Supplementary data

10.1136/bmjophth-2021-000854.supp7 Supplementary data

### Responses to symptoms of scenario 3 (MK)

**Figure 2:**
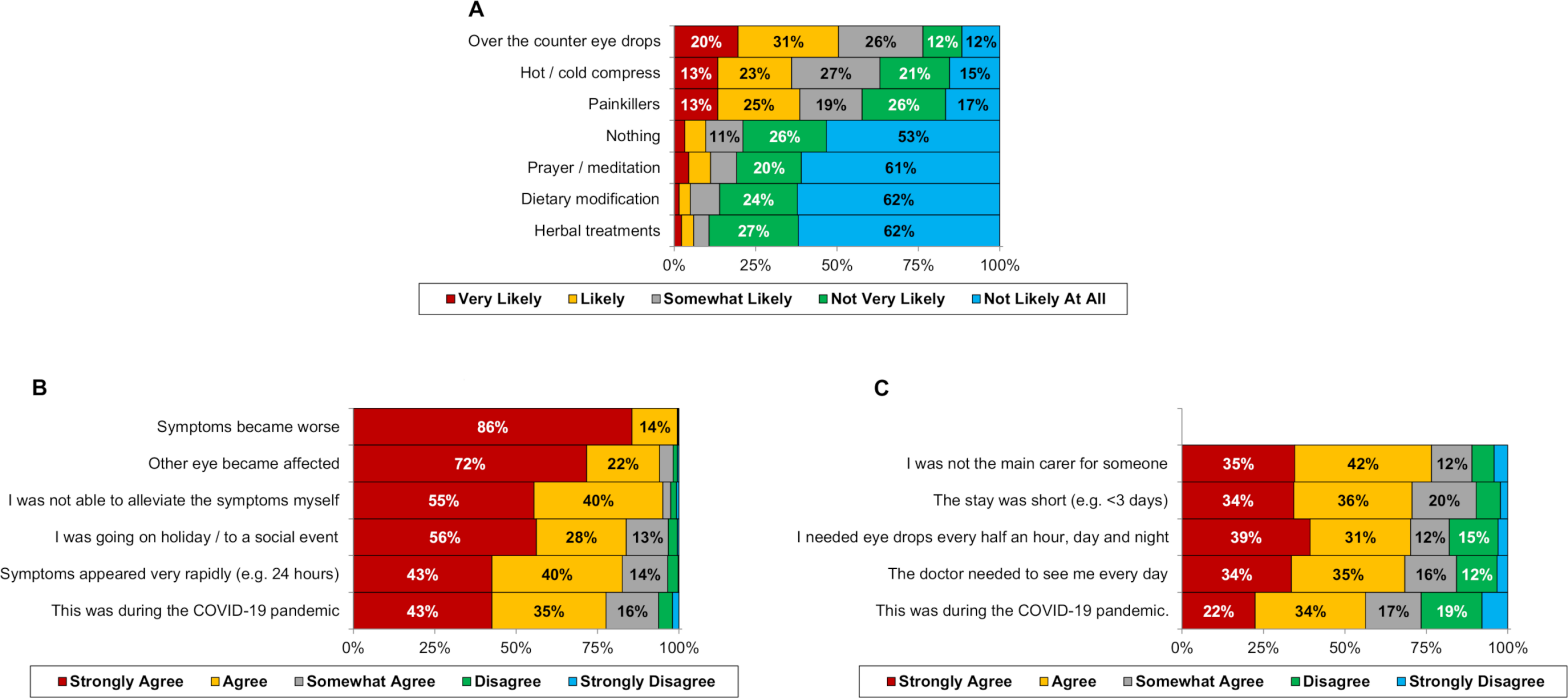
Participant responses to further questions relating to scenario 3 (microbial keratitis). For scenario 3 (microbial keratitis), participants were asked how likely they would be to use various self-management strategies (A), and how likely they would be to seek help (B) or agree to a hospital admission (C) in a range of situations. For both (B) and (C), Friedman’s test found significant differences in responses across the situations (both p<0.001). Unlabelled bars each consist of <10% of participants.

Participants were asked how they would react if they developed the symptoms of scenario 3 (MK). The first question related to self-management, with the preferred strategies being over-the-counter eye drops, painkillers and hot compress, reported as likely/very likely by 50.5%, 38.6% and 36.1%, respectively (figure 2A). Participants were also asked how likely they would be to seek medical attention for scenario 3 in a range of different situations (figure 2B). Of these, worsening of symptoms and spreading to the other eye elicited the strongest responses (strongly agree: 85.6% and 71.6%, respectively), with the COVID-19 pandemic being the lowest rated factor (strongly agree: 42.5%). Finally, participants were asked whether they would agree to hospital admission in a range of situations (figure 2C). Responses were generally similar for all situations, apart from the COVID-19 pandemic, for which participants were significantly less likely to agree to admission (p<0.001 for all pairwise comparisons).

### Sources of information about eye problems and COVID-19

A total of 358/392 (91.3%) participants indicated that they would seek information regarding eye problems before visiting an ophthalmologist. Of these, 319/358 (89.1%) completed the questions relating to their top three sources of information. The internet (211; 66.1%), a general practitioner (195; 61.1%) and an optometrist (148; 46.4%) were the most common (online supplemental figure 2A).

10.1136/bmjophth-2021-000854.supp8 Supplementary data

Regarding information sources about the COVID-19 pandemic (online supplemental figure 2B), for the 364 participants who completed this section, government briefings (262; 72.0%), the internet (235; 64.6%) and the TV/radio (205; 56.3%) were most frequently ranked within the top three sources. Comparisons by ethnicity found that white participants were significantly more likely to state that they use government briefings (75.0% vs 51.1%, p=0.002) or TV/radio (59.5% vs 37.8%, p=0.009) for information, as compared with non-white participants.

### Volunteering for research

**Figure 3:**
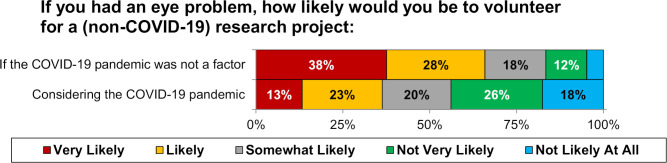
Participant willingness to be involved in ophthalmic research. Responses were found to differ significantly between questions (p<0.001 on Wilcoxon signed-rank test). Unlabelled bars each consist of <10% of participants.

If COVID-19 was not a factor, 65.9% of participants stated that they would be likely/very likely to volunteer for a research project. However, after considering the COVID-19 pandemic, this dropped to 36.3% (p<0.001, figure 3).

## Discussion

The COVID-19 outbreak has presented a far greater threat than other recent pandemics, for which the prevention of transmission is a very important management strategy. The ability of individuals to risk assess their needs is of the utmost importance to ensure adherence to transmission mitigation measures. The present study demonstrates the public’s ability to recognise the severity of ocular symptoms and seek medical attention accordingly; offers insight about the significance of various factors when deciding to seek help; and evidences the impact of the COVID-19 pandemic on reported healthcare-seeking behaviour and the decreased willingness of individuals to volunteer for non-COVID-19 research.

Appropriate healthcare-seeking behaviour is contingent on understanding one’s own health, which is influenced by profession, knowledge, social relationships and circumstances. Out of the conditions discussed in section III of the survey (eye disease, bowel cancer, angina and COVID-19), participants were most likely to state that they knew someone with eye disease. Although public knowledge of eye health has been found to be poor,^19–22^ this study’s participants were able to identify the increasing severity of the eye-related scenarios correctly. This supports the notion that knowledge of pathology is but one of the components in the process of seeking healthcare. Visual impairment was a differentiating symptom in the first three scenarios, and the commonality between scenarios 3 (MK) and 4 (painless loss of vision) that may explain the pattern of responses. Vision is considered as the most important sense,^35^ and its impairment is considered worse than heart disease.^18^ This was also echoed in these results, with scenarios 3 and 4 being reported by participants to be of similar severity, impact and urgency, and both were reported to be either similar to or worse than scenario 6 (chest pain).

Across all scenarios, the COVID-19 pandemic was associated with a significant reduction in the urgency of healthcare-seeking behaviour. Further investigation of scenario 3 (MK) also found the COVID-19 pandemic to be the factor that would make participants the least likely to seek urgent medical attention, or to agree to admission, in comparison with the other factors discussed in the survey. This illustrates the public’s concerns, and the potential altered healthcare-seeking behaviour that individuals may adopt, in view of the risks of attending healthcare services.^24 25 36^ The decrease in likelihood was more pronounced for seeking care than for admission to hospital, which may be due to the greater seriousness implicated by the need for admission, as well as it being the health professional’s suggestion. As such, apprehensions about engaging with medical services appear to have a considerable impact on the decision to seek help.

The disproportionately greater mortality and morbidity in ethnic minority groups^26^ have heightened health anxieties in these individuals,^28 29^ while the national lockdowns have worsened isolation, and compromised the public’s financial and personal well-being.^36–39^

In this sample, which mostly comprised white elderly retirees, greater age correlated with a greater perception of seriousness and urgency, particularly for the mild dry eye, bowel cancer and angina scenarios. These associations with age are curious, and likely reflect the higher prevalence of these conditions in these groups. Young UK citizens perceive eye disease as a concern for older life,^20^ hence younger individuals may be less inclined to seek healthcare and consider symptoms to be less severe. They may also rationalise symptoms, for example, chest pain on exertion to a non-cardiac cause. In this regard, the associations of younger age and lower perceived seriousness and urgency in the present study would be, to an extent, expected. However, due to the sample demographics (ie, the close association of younger age, non-white ethnicity and greater deprivation), it is difficult to discern the influence of these factors independently.

The relatively small non-white subgroup in this study was significantly more likely to know someone with COVID-19, and less likely to report the use of the government briefings as a main source of information about the pandemic. Greater deprivation, particularly in the non-white group, correlated with lower reported severity, impact and urgency of medical attention of select scenarios. These results are in agreement with other work describing the increased concerns and health anxiety in relation to the pandemic,^29 30 40^ and raise the possibility of altered healthcare seeking in these individuals. Although this study did not explore the specific work roles of participants, it is important to consider that individuals from greater deprivation may be less financially resilient, and the nature of their occupation may not permit working from home. Thus, differing beliefs and priorities reflecting their circumstance may influence their responses. The lower likelihood of using government briefings as a major source of information about the pandemic suggests this communication medium inadequately serves all demographic groups. The underlying reasons for these results are likely complex and, while the results from 49 non-white participants must be interpreted with caution, it is an indicator of the need for further consideration.

Healthcare information seeking is an integral component of healthcare-seeking behaviour.^41^ During this pandemic, alternate channels such as social media took a more prominent and frequently negative role in information dissemination.^42–45^ A tragic example from Iran in March 2020 occurred following social media posts about alcohol ingestion as a preventive measure, resulting in hundreds of deaths around the country.^46^ In the present study, the internet and clinical staff were the most preferred sources of information for eye symptoms, whereas the internet and traditional media (TV/radio/newspapers) were the most preferred for COVID-19, in keeping with other recent work.^47 48^ Social media did not feature highly, which is possibly related to the predominant elderly demographic of the study being less inclined to use social media. Effective utilisation of the internet for health information can be challenging.^49–51^ Lower proficiency with the internet and related technologies is associated with increased susceptibility to misinformation.^52 53^

Ophthalmic emergency services have been among the most disrupted around the globe as a result of the pandemic.^3–11^ MK is the most common non-surgical ophthalmic emergency requiring admission. Even with successful treatment of the acute infection, postinfective scarring can lead to permanent visual impairment, meaning that early initiation of treatment is critical for preserving vision. Although the feature composition of scenario 3 was typical of MK, real-world symptomatology may vary, particularly in the early stages of the disease where symptoms may be more akin to scenarios 1 and 2, or to other conditions predisposing MK. In keeping with the Rosenstock’s health belief model,^17^ this study demonstrates that milder symptoms are perceived to require medical attention less urgently, particularly during the pandemic. In the case of innocuous conditions such as dry eye, this behavioural adaptation may help decrease the transmission rate of COVID-19. However, in the event of a predisposing pathology or early MK, a delay that permits the disease to progress may lead to increased severity of MK by the time of presentation and, consequently, poorer final outcome. Such a phenomenon has been reported in patients with retinal detachments in this pandemic,^8^ as well as other hospital services that are being used less,^54 55^ and have patients presenting later with more severe disease.^56^ This warns us of how a ‘Swiss-cheese’ model of accident causation^57^ might arise in patients with early and mild symptoms of MK who, due to concerns regarding the pandemic, delay presentation and consequently have worse disease and final outcomes.

Public health literacy is vital to combat the pandemic. Scenarios 3–6 were all sight-threatening or life-threatening conditions, yet still 1%–6% of participants did not consider them serious, impactful or urgent. As such, accurate information about the pandemic and increasing awareness of eye health must remain important public health priorities.

The main strength of the current study is the relatively large sample size, comprising participants spread across England and with a range of socioeconomic backgrounds. In addition, the data were collected prospectively using a standardised and well-refined survey. However, the study also had several limitations, which largely resulted from the challenges of investigating public perceptions during a pandemic. Barriers to public engagement, in particular face-to-face interactions, meant that it was only feasible to collect data based on a convenience sampling approach using an online questionnaire. This was distributed using several channels to maximise its reach and with a view to including a diverse range of participants. However, despite this, there was a preponderance of participants within the areas surrounding the University of Birmingham and BMEC. In addition, the average age of participants was relatively high, and correlations between demographic factors were observed, with those of white ethnicity tending to be considerably older and less deprived than non-white participants. As such, the demographics of the included participants may not be the optimal representation of the UK as a whole; hence, the generalisability of the findings cannot be guaranteed. In addition, the observed associations between age, ethnicity and deprivation make it difficult to isolate the effect of these factors; hence, the observed effects of each of these on participants’ views may be confounded by other factors. Finally, the use of an online survey precluded participants who either did not have internet access or were not computer literate, which may have introduced selection bias, particularly for the questions relating to preferred sources of information.

As a result, future work should aim to investigate the demographics less well represented in the current study, for example, by targeting the promotion of the survey using communication media more used by these demographics, and adopting a purposive sampling methodology. This would yield more generalisable results and would help validate the current findings. In addition, the current study was only intended to identify the beliefs of the participants, not to explore the underlying reasons for why these beliefs were held. A future study that further investigated the reasoning behind participants’ beliefs would help further explain the findings of the current study, as well as potentially highlighting areas that could be targeted in future to disseminate heath education. Such a study would need to collect more detailed and qualitative responses, which would likely require a different format of investigation (eg, by telephone or face-to-face structured interviews).

In conclusion, the results of this study offer insight into the healthcare-seeking attitudes adopted by the public during the lockdown period. Highlighted here is the importance of accurate health information and adequate public education, so that individuals may risk assess their own needs and act accordingly. Continually assessing the public’s understanding of health campaigning is useful for decision makers. COVID-19 exacerbates the gap in health inequality and raises concerns about safely accessible healthcare. The case for large-scale lockdown is compelling; however, the implications of this behavioural adaptation must be carefully considered by policymakers to avoid potential deleterious consequences. Following on from this work, clinical departments are encouraged to audit their services to investigate the extent of local impact with regard to patient outcomes.

## Data Availability

Data are available upon reasonable request.
