# Supplementary material for: Public perceptions of eye symptoms and hospital services during the first UK lockdown of the COVID-19 pandemic: a web survey study
Source: BMJ Open Ophthalmol. 2021 Oct 13;6(1):e000854. doi: 10.1136/bmjophth-2021-000854 (PMC8520595; doi:10.1136/bmjophth-2021-000854)
Supplement: Supplementary data [file bmjophth-2021-000854supp002.pdf]

Confidential

Public perceptions of eye symptoms and hospital services during the COVID-19 pandemic

Thank you for taking part in this survey!

After this page (about you) there are 4 sections which, in total, should take less than 10 minutes to complete.

Age (years)	◯ 18-25<br>◯ 26 -35<br>◯ 36 - 45<br>◯ 46- 55<br>◯ 56 - 65<br>◯ 66-75<br>◯ 76 +<br>◯ Prefer not to say
Gender<br><br>Which of the following describes how you see yourself?	◯ Male<br>◯ Female<br>◯ Another way<br>◯ Prefer not to say
Employment<br><br>Please indicate your current employment situation.	◯ In education either full-time or part-time<br>◯ Full time employed or self employed<br>◯ Part-time employed or self employed<br>◯ Retired<br>◯ Homemaker<br>◯ Not working due to illness or disability<br>◯ Unemployed
Ethnicity<br><br>Please read the following list and tick the appropriate box that you feel best describes your ethnic origins.	◯ White<br>◯ Black or Black British<br>◯ Asian or Asian British<br>◯ Mixed<br>◯ Other<br>◯ Prefer not to say
What is your full postcode?	______________________________

Butt GF, *et al. BMJ Open Ophth* 2021; 6:e000854. doi: 10.1136/bmjophth-2021-000854

Section I

Below you will find 6 short scenarios describing symptoms that occur with a number of different diseases.

Please read each scenario carefully and imagine that you are actually experiencing these symptoms.

After each scenario there are a few questions to answer.

Confidential

1 a) Scenario 1

Over the previous week you have noted that your right eye feels gritty as though you have sand in it. The eye looks minimally red, it is not sticky, and your vision is unaffected. You have not experienced this symptom before.

	Very	Moderately	Somewhat	Not very	Not at all
How SERIOUS would you say these symptoms are?	◯	◯	◯	◯	◯
How IMPACTFUL would these symptoms be on your daily life (i.e. your day to day activities)?	◯	◯	◯	◯	◯
How QUICKLY would you seek medical attention for these symptoms if the COVID-19 pandemic was NOT a factor?	◯	◯	◯	◯	◯
How QUICKLY would you seek medical attention for these symptoms taking into consideration the COVID-19 pandemic?	◯	◯	◯	◯	◯

Butt GF, *et al*. *BMJ Open Ophth* 2021; 6:e000854. doi: 10.1136/bmjophth-2021-000854

Confidential

1 b) Scenario 2

Over the previous week your right eye is red and sticky. It is slightly uncomfortable and your vision is slightly blurred but not all the time. You have not experienced these symptoms before.

	Very	Moderately	Somewhat	Not very	Not at all
How SERIOUS would you say these symptoms are?	○	○	○	○	○
How IMPACTFUL would these symptoms be on your daily life (i.e. your day to day activities)?	○	○	○	○	○
How QUICKLY would you seek medical attention for these symptoms if the COVID-19 pandemic was NOT a factor?	○	○	○	○	○
How QUICKLY would you seek medical attention for these symptoms taking the COVID-19 pandemic into consideration?	○	○	○	○	○

Butt GF, *et al. BMJ Open Ophth* 2021; 6:e000854. doi: 10.1136/bmjophth-2021-000854

Confidential

1 c) Scenario 3

Over the previous 2 days your right eye is red, painful and sensitive to light. It is sticky and your vision is blurred. You also notice there is a white area on your eye. You have not experienced these symptoms before.

	Very	Moderately	Somewhat	Not very	Not at all
How SERIOUS would you say these symptoms are?	○	○	○	○	○
How IMPACTFUL would these symptoms be on your daily life (i.e. your day to day activities)?	○	○	○	○	○
How QUICKLY would you seek medical attention for these symptoms if the COVID-19 pandemic was NOT a factor?	○	○	○	○	○
How QUICKLY would you seek medical attention for these symptoms taking the COVID-19 pandemic into consideration?	○	○	○	○	○

Butt GF, *et al. BMJ Open Ophth* 2021; 6:e000854. doi: 10.1136/bmjophth-2021-000854

Confidential

1 d) Scenario 4

Over the previous day the vision in your right eye becomes very blurred. The eye is NOT red, painful or sticky. You have not experienced these symptoms before.

	Very	Moderately	Somewhat	Not very	Not at all
How SERIOUS would you say these symptoms are?	○	○	○	○	○
How IMPACTFUL would these symptoms be on your daily life (i.e. your day to day activities)?	○	○	○	○	○
How QUICKLY would you seek medical attention for these symptoms if the COVID-19 pandemic was NOT a factor?	○	○	○	○	○
How QUICKLY would you seek medical attention for these symptoms taking the COVID-19 pandemic into consideration?	○	○	○	○	○

Butt GF, *et al*. *BMJ Open Ophth* 2021; 6:e000854. doi: 10.1136/bmjophth-2021-000854

1 e) Scenario 5

Over the previous week you visit the bathroom and notice that there is blood in your stools. This has happened several times over the last couple of weeks. Recently you've been going to the toilet more often and have had some diarrhoea. You have also noticed that you have been losing weight, which is unusual because your appetite has been normal, and you have not been exercising more than normal. You are also feeling run down and very tired.

	Very	Moderately	Somewhat	Not very	Not at all
How SERIOUS would you say these symptoms are?	○	○	○	○	○
How IMPACTFUL would these symptoms be on your daily life (i.e. your day to day activities)?	○	○	○	○	○
How QUICKLY would you seek medical attention for these symptoms if the COVID-19 pandemic was NOT a factor?	○	○	○	○	○
How QUICKLY would you seek medical attention for these symptoms taking the COVID-19 pandemic into consideration?	○	○	○	○	○

1 f) Scenario 6

Over the previous week whenever you undertake physical activity you experience a pain across your chest. The pain feels like a heaviness and tightness in the chest area. You also experience light-headedness and a slight shortness of breath. The symptoms subside after a few minutes but start again when you engage in strenuous activities or when you experience emotional upset and stress.

	Very	Moderately	Somewhat	Not very	Not at all
How SERIOUS would you say these symptoms are?	○	○	○	○	○
How IMPACTFUL would these symptoms be on your daily life (i.e. your day to day activities)?	○	○	○	○	○
How QUICKLY would you seek medical attention for these symptoms if the COVID-19 pandemic was NOT a factor?	○	○	○	○	○
How QUICKLY would you seek medical attention for these symptoms taking the COVID-19 pandemic into consideration?	○	○	○	○	○

Butt GF, *et al. BMJ Open Ophth* 2021; 6:e000854. doi: 10.1136/bmjophth-2021-000854

Section II

In this section we will explore one set of symptoms in detail.

Butt GF, *et al. BMJ Open Ophth* 2021; 6:e000854. doi: 10.1136/bmjophth-2021-000854

Confidential

Your right eye is red, painful and sensitive to light. The eye is sticky and your vision is blurred. You also notice a white area on your eye. You have not experienced these symptoms before.

2 a) If you were to experience these symptoms, how likely is it that you would use the treatments or actions listed below to try and alleviate or improve them?

Please check the appropriate box for each treatment or action:

	Very likely	Likely	Somewhat likely	Not very likely	Not likely at all
Herbal treatments	○	○	○	○	○
Hot or cold compresses on the eyelids	○	○	○	○	○
Nothing	○	○	○	○	○
Over the counter eye drops / ointments e.g. artificial tears	○	○	○	○	○
Painkillers e.g. paracetamol, aspirin, ibuprofen	○	○	○	○	○
Prayer / meditation / relaxation / mindfulness	○	○	○	○	○
Dietary modification/change	○	○	○	○	○

Butt GF, *et al. BMJ Open Ophth* 2021; 6:e000854. doi: 10.1136/bmjophth-2021-000854

Confidential

2 b) The following statements refer to reasons why you might want to seek medical attention quickly if you experienced a red, painful, sticky eye with blurred vision.

I would seek medical attention quickly

	Strongly agree	Agree	Neither agree nor disagree	Disagree	Strongly disagree
If my symptoms appeared very rapidly e.g. 24 hours	○	○	○	○	○
If my other eye became affected	○	○	○	○	○
If I was not able to alleviate the symptoms myself	○	○	○	○	○
If my symptoms became worse	○	○	○	○	○
If I was going on holiday or to a social event I was heavily involved with	○	○	○	○	○
If this was during the COVID-19 pandemic	○	○	○	○	○

2 c) The eye doctor (ophthalmologist) has advised that you should be admitted to hospital to receive care for your red, painful, sticky eye with blurred vision.

The following statements are reasons that may affect your decision to be admitted.

I would AGREE to hospital admission

	Strongly agree	Agree	Neither agree nor disagree	Disagree	Strongly disagree
If I needed eye drops every half hour, through the day and night	○	○	○	○	○
If the doctor needed to see me every day	○	○	○	○	○
If I WAS NOT the main carer for someone	○	○	○	○	○
If the stay was short (e.g. less than 3 days)	○	○	○	○	○

Confidential

In the same scenario:

I would agree to hospital admission

	Strongly agree	Agree	Neither agree nor disagree	Disagree	Strongly disagree
If it was during the COVID-19 pandemic.	◯	◯	◯	◯	◯

Butt GF, *et al. BMJ Open Ophth* 2021; 6:e000854. doi: 10.1136/bmjophth-2021-000854

Section III

In this section you will answer some questions about how you use different sources of health information and your knowledge of certain conditions.

3 a) Information about eye problems.

If you were suffering with eye symptoms, would you seek information regarding your eye problem before you see an eye doctor (ophthalmologist)?

◯ Yes
◯ No

3 b) If you answered 'yes' to 3a please rank the TOP THREE sources of information that you would use before you see an eye doctor (ophthalmologist)?

Please rank the TOP THREE sources and leave the remainder blank.

	Main source of information	Second source	Third source
Books	○	○	○
I would not get any information	○	○	○
Internet	○	○	○
Medical or scientific literature	○	○	○
Newspaper / magazines	○	○	○
NHS Direct or other telephone helpline (e.g. 111)	○	○	○
Occupational Health at your place of work	○	○	○
Optometrist (Optician)	○	○	○
Pharmacist	○	○	○
Relatives / friends	○	○	○
Social Media (e.g. WhatsApp, Facebook, Twitter)	○	○	○
TV / Radio	○	○	○
Walk-in Health Centre	○	○	○
Work colleagues	○	○	○
Your General Practitioner (GP)	○	○	○

Confidential

3 c) Information about the COVID-19 pandemic.

What are your sources of information about the COVID-19 pandemic?

Please rank the TOP THREE sources and leave the remainder blank.

	Main source of information	Second source	Third source
Community leader / Religious leader	○	○	○
Government briefings	○	○	○
Internet	○	○	○
Medical or Scientific literature	○	○	○
Newspaper / Magazines	○	○	○
NHS Direct or other telephone helpline (e.g. 111)	○	○	○
Relatives / Friends	○	○	○
Someone who suffered from COVID-19	○	○	○
Social media (e.g. WhatsApp, Facebook, Twitter etc)	○	○	○
TV / Radio	○	○	○
Work colleagues	○	○	○

Butt GF, *et al. BMJ Open Ophth* 2021; 6:e000854. doi: 10.1136/bmjophth-2021-000854

3 d) Do you know or have you known anyone with the following conditions? Please indicate who this person is / these persons are (e.g. family, friend) by checking the relevant box(es) for each condition. 'Other' could include somebody you have looked after or seen with this condition if you are a healthcare worker. Check 'no-one' if you do not know anyone with the condition.

You may check more than one box.

	Friend(s)	Family	Other	No-one
Eye Disease	☐	☐	☐	☐
Bowel cancer	☐	☐	☐	☐
Angina (chest pain casued by heart disease)	☐	☐	☐	☐
COVID-19	☐	☐	☐	☐

Butt GF, *et al. BMJ Open Ophth* 2021; 6:e000854. doi: 10.1136/bmjophth-2021-000854

Section IV

Volunteering for research.

Clinical research is a fundamental part of progressing medical science. For example, without the help of patients volunteering, it would not be possible to discover and develop better treatments. This is true for all medical condtions including COVID-19.

Butt GF, *et al. BMJ Open Ophth* 2021; 6:e000854. doi: 10.1136/bmjophth-2021-000854

In this question we would like to know if you did have an eye problem how likely you are to volunteer for a NON-COVID-19 related research project on your eye condition (e.g. trial of a new drug or having blood samples taken that could require numerous extra visits to the hospital) and if the COVID-19 pandemic would affect your opinion about volunteering.

4 a) How likely are you to volunteer for a research project?

	Very likely	Likely	Somewhat likely	Not very likely	Not likely at all
If the COVID-19 pandemic WAS NOT a factor	○	○	○	○	○
Taking the COVID-19 pandemic into consideration	○	○	○	○	○
