# Supplementary material for: Public perceptions of eye symptoms and hospital services during the first UK lockdown of the COVID-19 pandemic: a web survey study
Source: BMJ Open Ophthalmol. 2021 Oct 13;6(1):e000854. doi: 10.1136/bmjophth-2021-000854 (PMC8520595; doi:10.1136/bmjophth-2021-000854)
Supplement: Supplementary data [file bmjophth-2021-000854supp003.pdf]

S3) Supplementary Figure 1 – Participant map

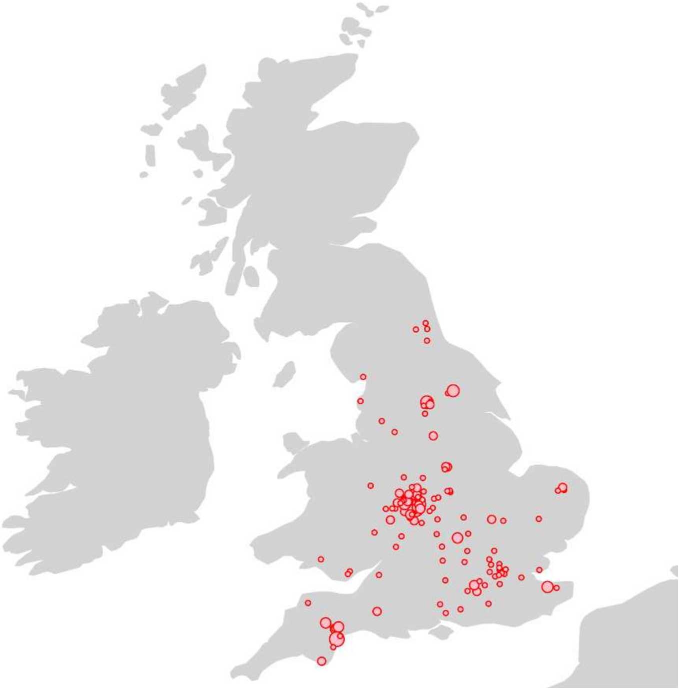

Participants who provided a valid postcode (N=371) were grouped based on their postcode district. Points are plotted at each of these postcode districts, with the size of the point representing the number of participants; the maximum number of participants within a district was N=40.

Participant map. The district component was extracted from all postcodes provided by participants, the longitude and latitude of which was extracted from https://www.doogal.co.uk/PostcodeDistricts.php. The R package "maps" was then used to generate a world map, which was cropped to only display the UK. The "ggplot2" package was then used to plot points representing each included postcode district onto this map. The size of these points represented the total numbers of participants within each district.
