# Supplementary material for: Public perceptions of eye symptoms and hospital services during the first UK lockdown of the COVID-19 pandemic: a web survey study
Source: BMJ Open Ophthalmol. 2021 Oct 13;6(1):e000854. doi: 10.1136/bmjophth-2021-000854 (PMC8520595; doi:10.1136/bmjophth-2021-000854)
Supplement: Supplementary data [file bmjophth-2021-000854supp004.pdf]

S3) Supplementary Table 1. Respondent characteristics by ethnicity

	Ethnicity		
	White	***Non-White***	**p-Value**
Age (Years)	*Mean: 65.1**	*Mean: 36.5**	**<0.001****
18-35	37 (10.6%)	32 (65.3%)	
36-65	74 (21.3%)	12 (24.5%)	
66+	237 (68.1%)	5 (10.2%)	
Gender			**0.039**
Male	121 (34.8%)	25 (51.0%)	
Female	227 (65.2%)	24 (49.0%)	
Employment			**<0.001**
Employed / In Education	102 (29.3%)	44 (89.8%)	
Unemployed / Retired	246 (70.7%)	5 (10.2%)	
Index of Multiple Deprivation [N=368]			**<0.001****
Decile 1-3 (Most Deprived)	53 (16.1%)	21 (53.8%)	
Decile 4-7	179 (54.4%)	11 (28.2%)	
Decile 8-10 (Least Deprived)	97 (29.5%)	7 (17.9%)	
Do You Know or Have Known Someone with the Following Conditions?			
Eye Disease	234 (67.2%)	29 (59.2%)	0.264
Bowel Cancer	194 (55.7%)	11 (22.4%)	**<0.001**
Angina	212 (60.9%)	20 (40.8%)	**0.009**
COVID-19	192 (55.2%)	38 (77.6%)	**0.003**
Sources of Information about Eye Problems [N=316]*			
Internet	180 (64.7%)	30 (78.9%)	0.099
General Practitioner	171 (61.5%)	23 (60.5%)	1.000
Optometrist (Optician)	139 (50.0%)	8 (21.1%)	**<0.001**
Sources of Information about COVID-19 [N=361]*			
Government Briefings	237 (75.0%)	23 (51.1%)	**0.002**
Internet	199 (63.0%)	33 (73.3%)	0.188
TV / Radio	188 (59.5%)	17 (37.8%)	**0.009**

*Analyses are based on N=397, unless stated otherwise. p-Values are from Fisher's exact tests, unless stated otherwise, and bold p-values are significant at p<0.05. *The mean age was estimated by assigning each participant to the midpoint of the age range that they had specified. **p-Value from Mann-Whitney U test, as the factor is ordinal. ***Only the most commonly reported sources for the cohort as a whole are reported, and rates represent the proportion of participants that ranked these in their top three sources.*

BMJ Publishing Group Limited (BMJ) disclaims all liability and responsibility arising from any reliance placed on this supplemental material which has been supplied by the author(s)
