# Supplementary material for: Public perceptions of eye symptoms and hospital services during the first UK lockdown of the COVID-19 pandemic: a web survey study
Source: BMJ Open Ophthalmol. 2021 Oct 13;6(1):e000854. doi: 10.1136/bmjophth-2021-000854 (PMC8520595; doi:10.1136/bmjophth-2021-000854)
Supplement: Supplementary data [file bmjophth-2021-000854supp005.pdf]

**S4) Supplementary Table 2. Full Likert data for participants views on scenarios**

| | How Serious are These Symptoms | How Impactful are These Symptoms on Daily Life | How Quickly Would You Seek Attention If COVID-19 was not a Factor? | How Quickly Would You Seek Attention Considering COVID-19? |
|---|---|---|---|---|
| **Scenario 1 - Eye mildly red and gritty** | | | | |
| **Very** | 13 (3.2%) | 30 (7.5%) | 57 (14.2%) | 35 (8.7%) |
| **Moderately** | 77 (19.2%) | 85 (21.1%) | 126 (31.3%) | 87 (21.6%) |
| **Somewhat** | 125 (31.1%) | 145 (36.1%) | 79 (19.7%) | 80 (19.9%) |
| **Not Very** | 165 (41.0%) | 121 (30.1%) | 106 (26.4%) | 140 (34.8%) |
| **Not at all** | 22 (5.5%) | 21 (5.2%) | 34 (8.5%) | 60 (14.9%) |
| **Scenario 2 - Eye red, sticky and blurred** | | | | |
| **Very** | 95 (23.6%) | 103 (25.6%) | 145 (36.1%) | 105 (26.1%) |
| **Moderately** | 162 (40.3%) | 161 (40.0%) | 155 (38.6%) | 133 (33.1%) |
| **Somewhat** | 113 (28.1%) | 105 (26.1%) | 63 (15.7%) | 91 (22.6%) |
| **Not Very** | 28 (7.0%) | 28 (7.0%) | 32 (8.0%) | 55 (13.7%) |
| **Not at all** | 4 (1.0%) | 5 (1.2%) | 7 (1.7%) | 18 (4.5%) |
| **Scenario 3 - Eye red, painful, photophobia, sticky, blurred, white spot** | | | | |
| **Very** | 299 (74.4%) | 283 (70.4%) | 315 (78.4%) | 264 (65.7%) |
| **Moderately** | 77 (19.2%) | 89 (22.1%) | 64 (15.9%) | 97 (24.1%) |
| **Somewhat** | 21 (5.2%) | 24 (6.0%) | 17 (4.2%) | 27 (6.7%) |
| **Not Very** | 3 (0.7%) | 4 (1.0%) | 3 (0.7%) | 9 (2.2%) |
| **Not at all** | 2 (0.5%) | 2 (0.5%) | 3 (0.7%) | 5 (1.2%) |
| **Scenario 4 - Painless loss of vision** | | | | |
| **Very** | 251 (62.4%) | 269 (66.9%) | 266 (66.2%) | 230 (57.2%) |
| **Moderately** | 107 (26.6%) | 97 (24.1%) | 104 (25.9%) | 102 (25.4%) |
| **Somewhat** | 37 (9.2%) | 29 (7.2%) | 21 (5.2%) | 46 (11.4%) |
| **Not Very** | 5 (1.2%) | 5 (1.2%) | 8 (2.0%) | 17 (4.2%) |
| **Not at all** | 2 (0.5%) | 2 (0.5%) | 3 (0.7%) | 7 (1.7%) |
| **Scenario 5 - Rectal bleeding** | | | | |
| **Very** | 305 (75.9%) | 200 (49.8%) | 308 (76.6%) | 273 (67.9%) |
| **Moderately** | 65 (16.2%) | 121 (30.1%) | 67 (16.7%) | 79 (19.7%) |
| **Somewhat** | 28 (7.0%) | 69 (17.2%) | 22 (5.5%) | 27 (6.7%) |
| **Not Very** | 1 (0.2%) | 10 (2.5%) | 3 (0.7%) | 19 (4.7%) |
| **Not at all** | 3 (0.7%) | 2 (0.5%) | 2 (0.5%) | 4 (1.0%) |
| **Scenario 6 - Chest pain** | | | | |
| **Very** | 251 (62.4%) | 209 (52.0%) | 258 (64.2%) | 234 (58.2%) |
| **Moderately** | 111 (27.6%) | 137 (34.1%) | 101 (25.1%) | 103 (25.6%) |
| **Somewhat** | 24 (6.0%) | 42 (10.4%) | 29 (7.2%) | 40 (10.0%) |
| **Not Very** | 13 (3.2%) | 12 (3.0%) | 10 (2.5%) | 15 (3.7%) |
| **Not at all** | 3 (0.7%) | 2 (0.5%) | 4 (1.0%) | 10 (2.5%) |
