# Supplementary material for: Public perceptions of eye symptoms and hospital services during the first UK lockdown of the COVID-19 pandemic: a web survey study
Source: BMJ Open Ophthalmol. 2021 Oct 13;6(1):e000854. doi: 10.1136/bmjophth-2021-000854 (PMC8520595; doi:10.1136/bmjophth-2021-000854)
Supplement: Supplementary data [file bmjophth-2021-000854supp007.pdf]

S6) Supplementary Table 4. Scenarios 1 to 6 correlation with demographic factors in non-white subgroup

	Age (Years)	Gender (Female)	Employment (Unemployed/Retired)	Index of Multiple Deprivation
**Scenario 1**				
How Serious are Symptoms	Rho: -0.02 (p=0.899)	Rho: 0.06 (p=0.669)	Rho: -0.11 (p=0.459)	Rho: 0.19 (p=0.243)
How Impactful are Symptoms	Rho: -0.08 (p=0.562)	Rho: 0.07 (p=0.638)	Rho: 0.04 (p=0.796)	Rho: 0.23 (p=0.158)
How Quickly (Non-COVID)*	Rho: 0.03 (p=0.836)	Rho: 0.00 (p=0.976)	Rho: 0.22 (p=0.121)	Rho: 0.19 (p=0.245)
How Quickly (COVID)*	Rho: 0.16 (p=0.283)	Rho: -0.16 (p=0.285)	**Rho: 0.29 (p=0.042)**	Rho: 0.22 (p=0.185)
**Scenario 2**				
How Serious are Symptoms	Rho: -0.21 (p=0.155)	Rho: 0.19 (p=0.197)	Rho: -0.11 (p=0.443)	Rho: 0.27 (p=0.103)
How Impactful are Symptoms	Rho: -0.13 (p=0.361)	Rho: 0.04 (p=0.794)	Rho: -0.12 (p=0.425)	**Rho: 0.34 (p=0.037)**
How Quickly (Non-COVID)*	Rho: -0.14 (p=0.323)	**Rho: 0.35 (p=0.013)**	Rho: 0.07 (p=0.626)	**Rho: 0.32 (p=0.046)**
How Quickly (COVID)*	Rho: -0.07 (p=0.609)	Rho: 0.03 (p=0.835)	Rho: 0.06 (p=0.680)	**Rho: 0.38 (p=0.017)**
**Scenario 3**				
How Serious are Symptoms	Rho: -0.11 (p=0.464)	Rho: -0.01 (p=0.966)	Rho: -0.14 (p=0.353)	**Rho: 0.36 (p=0.026)**
How Impactful are Symptoms	Rho: -0.09 (p=0.534)	Rho: 0.11 (p=0.448)	Rho: 0.18 (p=0.216)	**Rho: 0.41 (p=0.009)**
How Quickly (Non-COVID)*	Rho: -0.15 (p=0.318)	Rho: 0.15 (p=0.298)	Rho: -0.04 (p=0.789)	**Rho: 0.39 (p=0.014)**
How Quickly (COVID)*	Rho: -0.06 (p=0.668)	Rho: 0.07 (p=0.654)	Rho: -0.06 (p=0.681)	Rho: 0.24 (p=0.143)
**Scenario 4**				
How Serious are Symptoms	Rho: 0.24 (p=0.103)	Rho: -0.11 (p=0.435)	Rho: 0.32 (p=0.024)	**Rho: 0.33 (p=0.038)**
How Impactful are Symptoms	Rho: 0.18 (p=0.218)	Rho: -0.12 (p=0.408)	Rho: 0.27 (p=0.061)	Rho: 0.31 (p=0.052)
How Quickly (Non-COVID)*	Rho: 0.09 (p=0.534)	Rho: -0.13 (p=0.362)	**Rho: 0.29 (p=0.042)**	Rho: 0.15 (p=0.359)
How Quickly (COVID)*	Rho: 0.17 (p=0.247)	Rho: -0.23 (p=0.105)	Rho: 0.27 (p=0.064)	Rho: 0.13 (p=0.443)
**Scenario 5**				
How Serious are Symptoms	Rho: -0.01 (p=0.955)	Rho: -0.26 (p=0.072)	Rho: 0.19 (p=0.185)	Rho: 0.26 (p=0.115)
How Impactful are Symptoms	Rho: 0.20 (p=0.176)	Rho: -0.21 (p=0.144)	Rho: 0.26 (p=0.076)	**Rho: 0.34 (p=0.034)**
How Quickly (Non-COVID)*	Rho: 0.20 (p=0.176)	Rho: -0.20 (p=0.172)	**Rho: 0.29 (p=0.043)**	**Rho: 0.42 (p=0.008)**
How Quickly (COVID)*	Rho: 0.21 (p=0.156)	**Rho: -0.29 (p=0.042)**	Rho: 0.27 (p=0.062)	Rho: 0.27 (p=0.097)
**Scenario 6**				
How Serious are Symptoms	Rho: -0.03 (p=0.817)	Rho: -0.08 (p=0.603)	Rho: 0.08 (p=0.574)	**Rho: 0.38 (p=0.017)**
How Impactful are Symptoms	Rho: 0.03 (p=0.845)	Rho: -0.12 (p=0.408)	Rho: 0.10 (p=0.501)	**Rho: 0.43 (p=0.006)**
How Quickly (Non-COVID)*	Rho: 0.02 (p=0.872)	Rho: -0.12 (p=0.392)	Rho: 0.10 (p=0.497)	Rho: 0.25 (p=0.122)
How Quickly (COVID)*	Rho: 0.12 (p=0.418)	Rho: -0.16 (p=0.270)	Rho: 0.13 (p=0.378)	Rho: 0.25 (p=0.124)

Analysis was performed using Spearman's (Rho) correlation coefficients, with positive coefficients representing greater agreement with the question with increasing values of ordinal variables, or for the stated category relative to the reference for nominal variables. Only respondents of non-White ethnicity were included (N=49). Bold values are significant at p<0.05. The details of the questions are abbreviated, and are detailed in full in the text. *The question asked how quickly the respondent would seek medical attention for symptoms either if the COVID-19 pandemic was not a factor (Non-COVID), or taking this into consideration (COVID).

BMJ Publishing Group Limited (BMJ) disclaims all liability and responsibility arising from any reliance placed on this supplemental material which has been supplied by the author(s)
