# Supplementary material for: Public perceptions of eye symptoms and hospital services during the first UK lockdown of the COVID-19 pandemic: a web survey study
Source: BMJ Open Ophthalmol. 2021 Oct 13;6(1):e000854. doi: 10.1136/bmjophth-2021-000854 (PMC8520595; doi:10.1136/bmjophth-2021-000854)
Supplement: Supplementary data [file bmjophth-2021-000854supp008.pdf]

S7) Supplementary figure 2. Preferred sources of information on eye problems and the COVID-19 pandemic

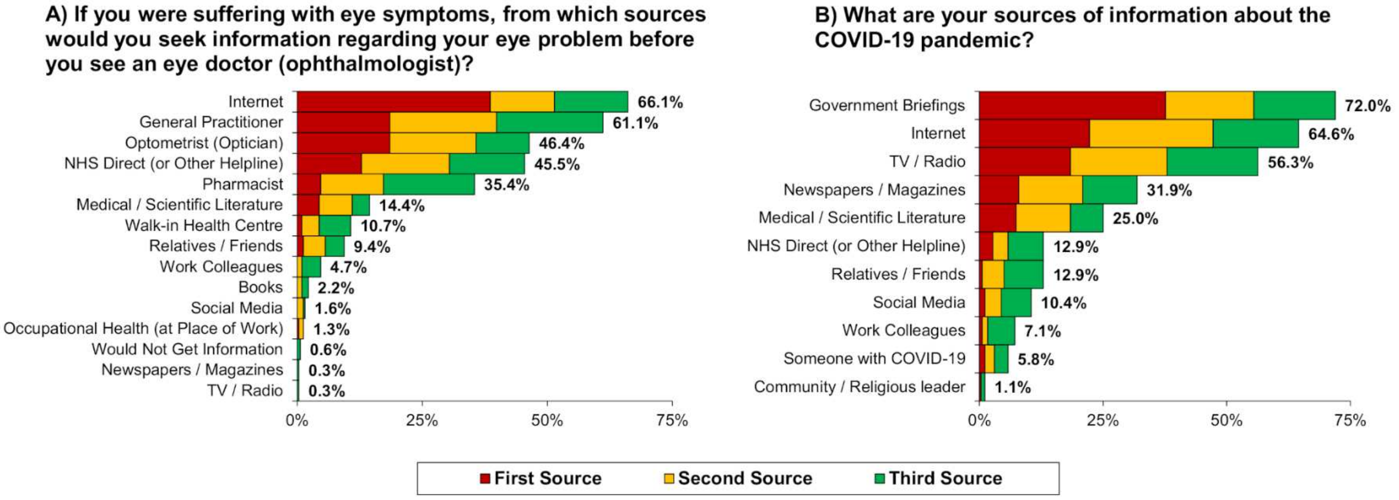

Plots are based on the N=319 and N=364 that answered the questions relating to eye problems and COVID-19, respectively. Percentages represent the proportion of participants that classified the stated information source as being within their top three.
